# Unidirectional superscattering by multilayered cavities of effective radial anisotropy

**DOI:** 10.1038/srep34775

**Published:** 2016-10-06

**Authors:** Wei Liu, Bing Lei, Jianhua Shi, Haojun Hu

**Affiliations:** 1 College of Optoelectronic Science and Engineering, National University of Defense Technology, Changsha, Hunan 410073, China

## Abstract

We achieve unidirectional forward superscattering by multilayered spherical cavities which are effectively radially anisotropic. It is demonstrated that, relying on the large effective anisotropy, the electric and magnetic dipoles can be tuned to spectrally overlap in such cavities, which satisfies the Kerker’s condition of simultaneous backward scattering suppression and forward scattering enhancement. We show that such scattering pattern shaping can be obtained in both all-dielectric and plasmonic multilayered cavities at different spectral positions, and believe that the mechanism we have revealed provides extra freedom for scattering shaping, which may play a significant role in many scattering related applications and also in optoelectronic devices made up of intrinsically anisotropic two dimensional materials.

With the rapid development of the field of metamaterials and metadevices^1^,^2^, recently the topic of scattering pattern manipulation based on the interferences of electric and artificial magnetic resonances has attracted a lot of attention (see refs 3, 4, 5 and references therein). The physical mechanism behind originates from the concept of Huygens source in the antenna theory^6^,^7^,^8^ and the proposal of Kerker^9^ to simultaneously suppress backward scattering and enhance forward scattering, which has only recently been specifically demonstrated in both all-dielectric and plasmonic nanostructures (for both dipolar and higher order modes)^4^,^8^,^10^,^11^,^12^,^13^,^14^,^15^,^16^,^17^,^18^,^19^,^20^,^21^,^22^. Moreover, the scattering suppression based on resonance interferences has been extended from the backward direction to other scattering angles^4^,^23^,^24^,^25^, the principle of which can be applied to generalize the concept of Brewster angle^24^,^25^.

In various nanostructures, usually resonances of the lowest order (dipolar resonances) are most accessible, which can be excited with high efficiency and thus are dominant. As a result, the achievement of the backward scattering suppression and forward scattering enhancement replying on overlapping electric dipoles (EDs) and magnetic dipoles (MDs) (the so called Kerker’s condition^9^,^26^) is still one of most outstanding examples of scattering pattern manipulations based on resonance interferences^3^,^12^,^13^,^17^,^18^. It is shown that even for homogeneous dielectric spheres, the EDs and MDs can be tuned to overlap partly, leading to unidirectional forward scattering^13^,^17^,^18^. Nevertheless, for simple homogeneous dielectric spheres, the EDs and MDs can not be tuned to resonantly overlap (their central resonant positions do not coincide) and thus the overlapping position does not locate at the resonance centre. Consequently, though the backward scattering has been significantly suppressed, the forward scattering is not strong enough to be in the superscattering regime^27^. Recently fully resonant overlapping of EDs and MDs and thus unidirectional forward superscattering has been achieved in core-shell plasmonic nanoparticles^4^,^12^,^28^,^29^, in dielectric nanodisks^19^ and in spheroidal dielectric nanoparticles^22^. For the latter two cases however, the scattering is dependent on the polarization and incident angle. Alternatively, it is recently proposed that even for homogeneous dielectric spheres, that electric radial anisotropy can be employed to enable resonant overlapping of EDs and MDs^30^. The problem is that the anisotropy required is too large to be found in naturally accessible materials.

In this work it is shown that multilayered cavities made up of isotropic layers can provide effective radial anisotropy that is large enough to enable resonant overlapping of EDs and MDs excited. Here we investigate the scattering of multilayered spherical resonators and demonstrate that in both all-dielectric and plasmonic cavities the Kerker’s condition can be fulfilled at different spectral positions, and thus significant backward scattering suppression and forward superscattering can be simultaneously achieved. We note that the mechanism we have revealed is general, and can be widely applied to cylindrical multilayered cavities and cavities of other shapes, to resonances of higher orders, and to other types of anisotropy^31^,^32^. We believe that such principle provides an extra dimension of freedom for resonance control and scattering shaping, which may shed new light to many scattering related applications, and to optoelectronic devices incorporating two dimensional materials that are intrinsically highly anisotropic.

## Results

### Theoretical analysis on plane wave scattering by multilayered cavities of effective radial anisotropy

**Figure 1:**
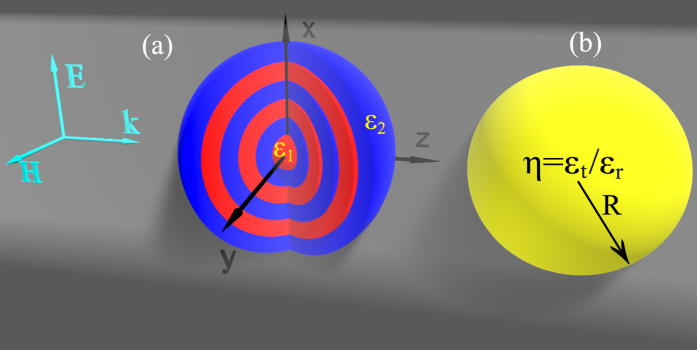
(**a**) Scattering of an *x* direction polarized (in terms of electric field) plane wave by a multilayered spherical cavity consisting of alternating isotropic layers of permittivities *ε*_1_ and *ε*_2_. The filling factor of the layer of permittivity *ε*_1_ is *f* in terms of overall layer width. The overall resonator radius is *R*. (**b**) A homogeneous radially anisotropic sphere of radius *R* and with radial permittivity *ε*_*r*_ and transverse permittivity *ε*_*t*_. The radial anisotropy parameter is defined as *η* = *ε*_*t*_/*ε*_*r*_ and all the materials involved are nonmagnetic.

Figure 1(a) shows the scattering configuration we study: the incident plane wave is polarized along *x* direction and the multilayered spherical resonator (overall radius *R*) is made of nonmagnetic alternating isotropic layers of two permittivity parameters *ε*_1_ and *ε*_2_ (*ε*_1_ ≠ *ε*_2_). When each individual layer width is far smaller then the effective wavelength of the incident wave in the layer, according to the the effective medium theory^33^,^34^,^35^,^36^, the layered resonator in Fig. 1(a) can be approximated as a homogeneous radially anisotropic sphere shown in Fig. 1(b) of the same radius *R*. The corresponding permittivity along radial and transverse directions are respectively:


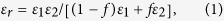



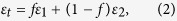


where *f* is the filling factor of the *ε*_1_ layer in terms of layer width. The anisotropy parameter *η* is defined as:


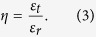


The scattering of plane waves by radially anisotropic spherical particles (both single layered or multilayered) can be solved analytically through generalized Mie theory^30^,^37^,^38^,^39^. The total scattering and absorption cross sections normalized by 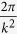 (*k* is the angular wave number in the background, which is vacuum in this work) are respectively:


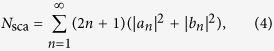



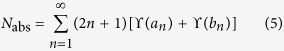


where the function ϒ(·) is defined as ϒ(·) = Re(·) − |·|^2^, and Re(·) means to adopt the real part; *a*_*n*_ and *b*_*n*_ are Mie scattering coefficients, which correspond to electric and magnetic resonance of the *n*–*th* order respectively (more specifically *a*_1_ and *b*_1_ correspond to ED and MD respectively). It is worth mentioning that *b*_*n*_ is dependent on *ε*_*t*_ only as the magnetic resonances are intrinsically transverse electric and thus are *ε*_*r*_ independent (thus *b*_*n*_ is not directly dependent on *η*); while in contrast *a*_*n*_ is dependent on both *ε*_*t*_ and *ε*_*r*_ and thus is directly *η*-dependent^30^,^37^,^38^,^39^. Both *a*_*n*_ and *b*_*n*_ can be analytically calculated, and for the simplest case of homogeneous sphere shown in Fig. 1(b) we have:


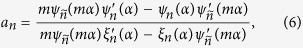



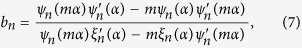


where 
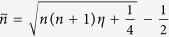
; *α* = *kR*; *ψ* and *ξ* are Riccati-Bessel functions^37^,^38^. When *η* = 1, 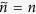 and Eqs (6 and 7) will be reduced to the well known expressions of isotropic spheres^37^,^38^. According to Eq. (4) the normalized scattering cross section contributed by ED and MD are respectively:


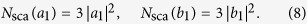


At the same time the law of energy conservation requires that^27^:


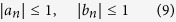


which together with Eq. (8) immediately requires that the upper limit of the normalized scattering cross section for both ED and MD are 3. Basically when dipolar resonance are dominantly excited, to be in the superscattering regime requires that *N*_sca_ > 3. When both *a*_*n*_ and *b*_*n*_ have been obtained, the far-field scattering patterns can be calculated directly^30^,^37^,^38^ (see the section of Methods).

### Unidirectional superscattering by all-dielectric multilayered cavities of *η* > 1

We start with all-dielectric multilayered cavities of *ε*_1_, *ε*_2_ > 0. It is easy to prove that:


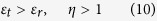


and the highest anisotropy parameter that can be achieved is


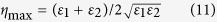


when *f* = 1/2. It is shown that the radial anisotropy can be employed to tune the resonant positions of EDs, which can enable the fully resonant overlapping of EDs and MDs, and result in unidirectional forward superscattering^30^. For *η* > 1 though EDs can not be engineered to overlap with MDs of the same mode number (a series of EDs and MDs can be excited at different wavelengths, and a mode number is adopted to differentiate them; for example the resonance excited at the largest wavelength has a mode number 1^30^), it is possible to overlap EDs and MDs of different mode numbers.

**Figure 2:**
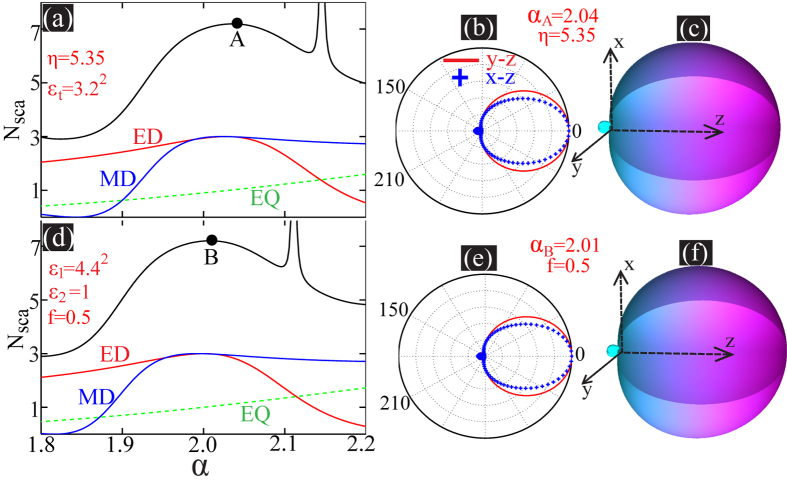
(**a**) Normalized scattering cross section spectra for the homogeneous radially anisotropic sphere, where both total scattering spectra (black curve, as is the case throughout the paper) and those contributed by ED (red curve), MD (blue curve), and EQ (dashed green curve) are shown. The specific parameters for the sphere are: *ε*_*t*_ = 3.2^2^ and *η* = 5.35. The overlapping resonant point is A (*α*_*A*_ = 2.04) and at this point the 2D and 3D scattering patterns are shown in (**b**) (red line and blue crosses indicate angular scattering intensities on the *y*–*z* and *x*–*z* panes respectively) and (**c**) respectively. (**d**) Normalized scattering cross section spectra for the multilayered cavity made up of 30 layers with alternating *ε*_1_ = 4.4^2^ and *ε*_2_ = 1 layers of the same width of *d* (*d* = *R*/30). The overlapping resonant point is B (*α*_*B*_ = 2.01) and at this position the 2D and 3D scattering patterns are shown in (**e**,**f**) respectively.

For all-dielectric structures where the material dispersion can be neglected, the scattering properties are fully scalable and thus are dependent only on the size parameter *α*. In Fig. 2(a) we show the scattering spectra of a homogeneous anisotropic sphere of *ε*_*t*_ = 3.2^2^ and *η* = 5.35, where both total scattering spectra (black curve, as is the case throughout the paper) and those contributed by ED and MD are shown. It is clear that ED and MD are resonantly overlapped (here the mode number for ED and MD are 1 and 2 respectively) and the resonant position is indicated by point *A* of *α*_*A*_ = 2.04. At point A it is certainly superscattering as the total scattering is almost twice the single channel limit^27^. Moreover, the overlapping of ED and MD will significantly suppress the backward scattering and enhance the forward scattering, which is shown in Fig. 2(b) [two-dimensional (2D) scattering patterns on the *x*–*z* and *y*–*z* planes] and Fig. 2(c) [full three-dimensional (3D) scattering pattern]. We note here that according to Fig. 2(b,c) the backward scattering has not been fully eliminated and the scattering patterns are not azimuthally symmetric (scattering patterns are not identical on different scattering planes containing *z* axis). This is because at point A, despite the resonant excitations of ED and MD, the electric quadrupole (EQ) has been also excited and is noneligible though the magnitude is much smaller (shown as the dashed green curve) in Fig. 2(a). The contributions of other multipoles (such as magnetic quadrupole, electric octupole and so on) are negligible at this overlapping resonant position (not shown).

The anisotropy parameter discussed above can be realized by an all-dielectric multilayered cavity of *ε*_1_ = 4.4^2^ and *ε*_2_ = 1, which effectively makes *ε*_*t*_ ≈ 3.2^2^, and *η* ≈ 5.35 when *f* = 1/2. In Fig. 2(d) we show the scattering spectra of a multilayered cavity consisting of 15 units: each unit is made up of two layers of the same width *d* 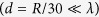 and the permittivity is *ε*_1_ = 4.4^2^ and *ε*_2_ = 1 respectively, and thus *f* = 0.5 and overall radius of the cavity is *R*. It is clear that the results agree well with those shown in Fig. 2(a) [the spectra is overall a bit red-shifted, which can be made convergent to those shown in Fig. 2(a) with decreasing layer width when the effective medium theory is more accurate], justifying the validity of the effective medium theory. The resonant overlapping position is indicated by point B of *α*_*B*_ = 2.01, and the corresponding scattering patterns are shown in Fig. 2(e,f), which also agree well with those obtained through effective medium theory shown in Fig. 2(b,c), and confirms the unidirectional superscattering of the all-dielectric multilayered cavity.

### Unidirectional superscattering by plasmonic multilayered cavities of *η* < 1

**Figure 3:**
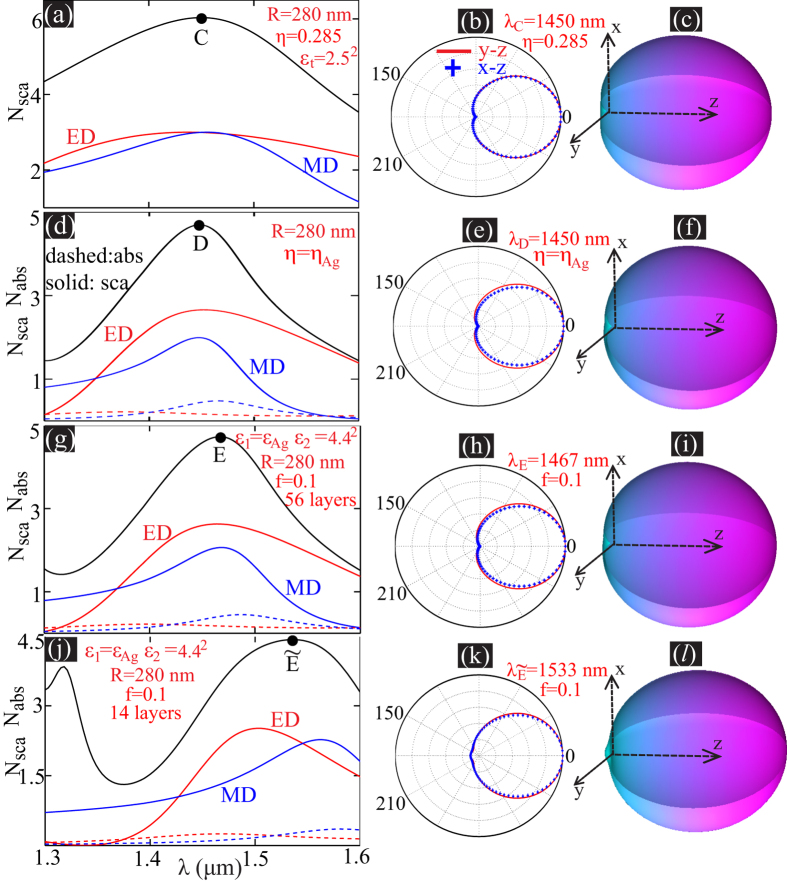
(**a**) Normalized scattering cross section spectra for a homogeneous radially anisotropic sphere: *R* = 280 nm, *ε*_*t*_ = 2.5^2^ and *η* = 0.285. The overlapping resonant point is C (*λ*_*C*_ = 1450 nm) and at this point the 2D and 3D scattering patterns are shown in (**b**,**c**) respectively. (**d**) Normalized scattering (solid curves) and absorption (ED: red dashed; MD: blue dashed) cross section spectra for a homogeneous radially anisotropic sphere of *R* = 300 nm, for which the effective parameters are obtained through Eqs (1, 2, 3) with *ε*_1_ = *ε*_*Ag*_, *ε*_2_ = 4.4^2^ and *f* = 0.1. The dispersive anisotropy parameter obtained is *η* = *η*_Ag_. The overlapping resonant point is D (*λ*_*D*_ = 1450 nm) and the scattering patterns are shown in (**e**,**f**). (**g**) The scattering and absorption spectra for the multilayered cavity made up of 56 layers with alternating silver and dielectric (*ε*_2_ = 4.4^2^) layers of width 1 nm and 9 nm respectively. The overlapping resonant point is E (*λ*_*E*_ = 1467 nm) and the scattering patterns are shown in (**h**,**i**). (**j**) The scattering and absorption spectra for the multilayered cavity made up of 14 layers with alternating silver and dielectric layers of width 4 nm and 36 nm respectively. The resonant point is 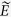 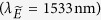 and the scattering patterns are shown in (**k**,**l**).

To achieve unidirectional superscattering relying on overlapping EDs and MDs of the same mode number requires *η* < 1. In Fig. 3(a) we show the scattering spectra of a homogeneous anisotropic sphere of *R* = 280 nm, *ε*_*t*_ = 2.5^2^ and *η* = 0.285, where ED and MD of the same mode number of 1 (ED and MD excited at the largest wavelength) are resonantly overlapped and the resonant position is *C* (*λ*_*C*_ = 1450 nm). At this point it is unidirectional superscattering as proved by the scattering patterns shown in Fig. 3(b,c).

The condition of *η* < 1 can be satisfied in hybrid plasmonic-dielectric multilayered cavities of *ε*_1_ < 0 and *ε*_2_ > 0 according to Eqs (1, 2, 3). For a multilayered cavity of *ε*_*t*_ = 2.5^2^ and *η* = 0.285, it requires that *ε*_1_ ≈ −112 and *f* = 0.1 if *ε*_2_ = 4.4^2^. We can design a silver-dielectric (*ε*_2_ = 4.4^2^) hybrid cavity of *f* = 0.1 and *R* = 280 nm, where the permittivity of silver (*ε*_*Ag*_) is taken from experimental data^40^ (see the supplementary information for more details). Of such a plasmonic cavity though the effective parameters (*ε*_*t*_, *ε*_*r*_ and *ε*_Ag_) are dispersive, the resonant overlapping of ED and MD poses only the requirement that the anisotropy parameter of *η* = 0.285 is provided at the resonant position, which silver can meet [*Re*(*ε*_*Ag*_) ≈ −112 when *λ* = 1450 nm]. In Fig. 3(d) we show both the scattering and absorption spectra of a homogeneous sphere of effective parameters obtained through Eqs (1, 2, 3) with *ε*_1_ = *ε*_*Ag*_ (complicated when loss of silver is considered), *ε*_2_ = 4.4^2^ and *f* = 0.1. The resonant position is *D* (*λ*_*D*_ = 1450 nm), where the ED and MD are resonantly overlapped. In contrast to the results in Fig. 3(a), the ED and MD are not of the same scattering magnitude due to the effect of Ohmic losses of silver, which lead to different absorptions of ED and MD (see the dashed absorption curves). Nevertheless, as is shown in Fig. 3(e,f), unidirectional superscattering with negligible backward scattering can still be achieved though the pattern is not rigourously azimuthally symmetric.

Now we turn to the multilayered plasmonic cavity consisting of 28 units: each unit is made up of a silver layer of width 1 nm and a isotropic dielectric layer (*ε*_2_ = 4.4^2^) of width 9 nm, and thus *f* = 0.1 and overall radius of the cavity *R* = 280 nm. In Fig. 3(g) we show the scattering and absorption spectra of such a multilayered cavity. The results agree well with those shown in Fig. 3(d) obtained through effective medium theory. The resonant overlapping position is indicated by point E of *λ*_*E*_ = 1467 nm, and the corresponding scattering patterns are shown in Fig. 3(h,i), which confirms the unidirectional superscattering of the multilayered plasmonic cavity.

It is worth mentioning that here for the results shown Fig. 3(g–i) we neglect the nonlocal effect^41^,^42^ of the thin silver layer of 1 nm width. The study of the multilayered cavity with nonlocal effect is itself rather complicated, which would be even more challenging when the quantum effects are also present^43^,^44^. We leave such a problem to a future study. Nevertheless to verify the feasibility of our proposal, we study alternatively a multilayered plasmonic cavity consisting of 7 units: each unit is made up of a silver layer of width 4 nm (of this geometric size, the nonlocal effect is negligible^41^,^42^) and an isotropic dielectric layer (*ε*_2_ = 4.4^2^) of width 36 nm. Similarly the radius of the cavity would be *R* = 280 nm and the filling ratio *f* = 0.1. In Fig. 3(j) we show the scattering and absorption spectra and it is clear that they deviate significantly from those obtained through effective medium theory [see Fig. 3(d)]. This is understandable, as with increasing layer widths, the effectively medium theory would become less and less accurate. However, it is clear that the effective medium theory can still be employed as a useful guide, as is demonstrated in Fig. 3(j) where both ED and MD are efficiently excited and can partly overlap due to the effective radial anisotropy. This guarantees a good directionality of the forward scattering [see the 2D and 3D scattering patterns shown in Fig. 3(k,l) respectively]. Another effect we neglect here is the surface scattering effect of thin metal layer^45^, which would make the silver more lossy in the spectrum regime we have investigated. We show that though such effect would reduce the total scattering cross sections, it would not comprise significantly the feature of unidirectional forward scattering (see the supplementary information for more details).

**Figure 4:**
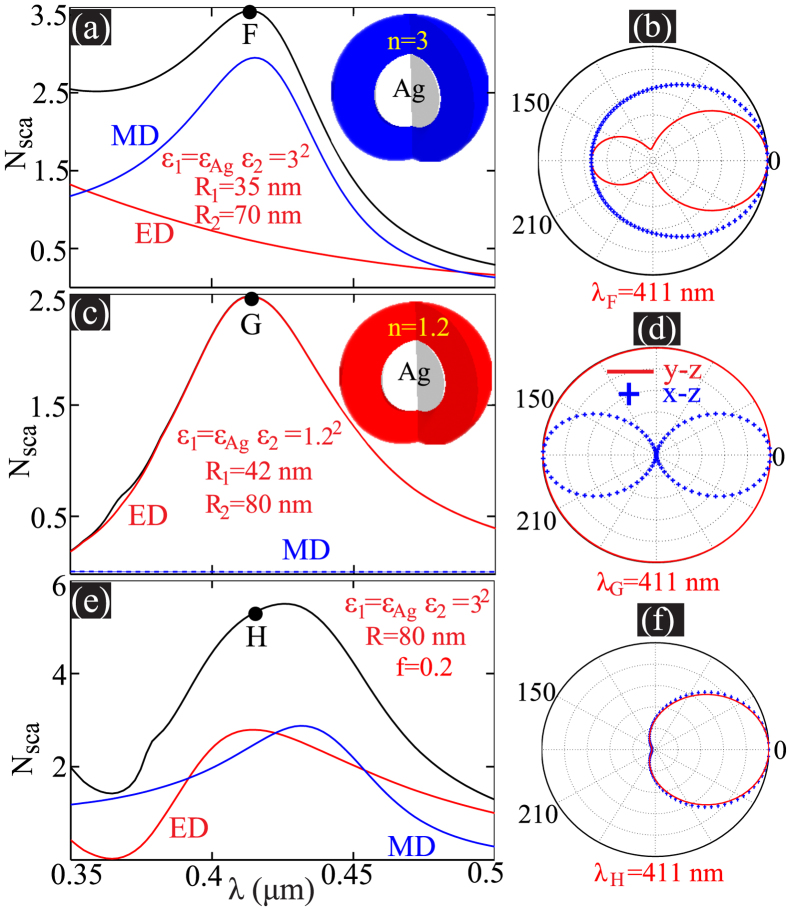
Normalized scattering cross section spectra of two layered silver-dielectric cavity of dielectric permittivity *ε*_2_ = 3, *R*_1_ = 35 nm, *R*_2_ = 70 nm in (**a**) and *ε*_2_ = 1.2, *R*_1_ = 42 nm, *R*_2_ = 80 nm in (**b**). The resonant positions are indicated by F and G (*λ*_*F*_ = *λ*_*G*_ = 411 nm), and at those points the 2D scattering patterns are shown in (**b**,**d**) respectively. (**e**) Normalized scattering cross section for the multilayered cavity made up of 8 layers with alternating silver and dielectric (*ε*_2_ = 3^2^) layers of width 4 nm and 16 nm respectively. At point H (*λ*_*H*_ = 411 nm) the 2D scattering patterns are shown in (**f**).

It is well known that in a simple two or three layered core-shell metal-dielectric cavity, the ED and MD can be tuned to resonantly overlap^12^,^28^,^46^, producing unidirectional forward superscattering^12^. Now the question is: why do we need the many layered plasmonic cavity described above to overlap EDs and MDs? Though it is shown specifically that through geometric tuning the resonant overlapping point of ED and MD can be pushed to the visible spectral regime in simple core-shell structures^46^, the problem is that in such structures it is extremely challenging to resonantly overlap ED and MD at wavelengths close to the plasmon frequency (at *λ* = 400 nm for example). To efficiently excite MDs at small wavelengths, it is known that high permittivity dielectric materials are usually required^47^,^48^. While high permittivity dielectric layer will red-shift the EDs, making them spectrally separated from MDs. In Fig. 4(a) we show the scattering spectra of a silver-dielectric (*ε*_1_ = 3^2^) core-shell cavity (inner radius *R*_1_ = 35 nm and outer radius *R*_2_ = 70 nm) where the MD resonates at point F (*λ*_*F*_ = 411 nm). However at this point the scattering magnitude of ED is much smaller, rendering significant backward scattering, as can be observed in the scattering patterns shown in Fig. 4(b). At the same time, with a low index dielectric layer, the EDs can be efficiently excited at small wavelength, while under such circumstance the MD can not be efficiently excited, making the scattering pattern a typical ED type with equal forward and backward scattering. This is demonstrated in Fig. 4(c) where we show the scattering spectra of a silver-dielectric (*ε*_1_ = 1.2^2^) core-shell resonator (*R*_1_ = 42 nm, *R*_2_ = 80 nm). Here the ED resonates at point G (*λ*_*G*_ = 411 nm), where however the MD excitation is negligible. As a result, the scattering is contributed only by ED and thus not unidirectional [see the scattering patterns shown in Fig. 4(d)].

The challenge of overlapping EDs and MDs at small wavelengths close to the plasmon frequency is not insurmountable for multilayered plasmonic cavities of effective radial anisotropy. we employ a multilayered plasmonic cavity consisting of 4 units: each unit is made up of a silver layer of width 4 nm and a isotropic dielectric layer (*ε*_1_ = 3^2^) of width 16 nm, and thus *f* = 0.2 and overall radius of the cavity *R* = 80 nm. In Fig. 4(e) we show the scattering spectra of such a plasmonic multilayered cavity. It is clear that ED and MD can still overlap partly. At point H (*λ*_*H*_ = 411 nm), though ED and MD are not of exactly the same magnitude, the backward scattering still has been significantly suppressed with forward unidirectional superscattering [see the scattering patterns shown in Fig. 4(f)].

## Conclusions and Discussions

To conclude, in this work we study the plane wave scattering by multilayered cavities that possess large effective radial anisotropy. In such cavities, relying on the effective radial anisotropy, the EDs and MDs can be tuned to resonantly overlap, which thus satisfies the Kerker’s condition and produces unidirectional forward superscattering. It is demonstrated that such scattering shaping can be realized in both all-dielectric and plasmonic multilayered cavities. Moreover it is shown that in plasmonic cavities of effective radial anisotropy, the EDs and MDs can be made to resonantly overlap at small wavelengths in the violet spectral regime, which is not accessible for simple two layered metal-dielectric core-shell resonators. We note here that in this work we have confined our studies to dipolar modes, and based on the same approach higher order modes can be made to overlap, which can produced more collimated forward superscattering^30^. Also, we have studied only the case of *η* > 0, and quite naturally hyperbolic cavities of *η* < 0 can be studied using the same method^33^,^34^. Moreover, the principle we have revealed is general, which is also applicable to resonators of other shapes and to other kinds of anisotropy such as magnetic anisotropy or the intrinsic huge anisotropy of 2D materials. Such mechanism of resonance control and scattering pattern shaping based on effective anisotropy can shed new light to many particle scattering problems and is quite promising for various applications in the fields of nanoantennas, solar cells, bio-sensing and so on.

## Methods

To obtain the effective permittivities (along both radial and transverse directions) and anisotropic parameters of both all-dielectric and plasmonic multilayered cavities, we apply the effective media theory [see Eq. (1)]. For all-dielectric structures, this theory will be valid as long as each dielectric layer width is far smaller than the effective wavelength. For plasmonic structures however, the excitation of plasmonic modes can made the effective wavelength extremely small, which can render the effective medium theory invalid in some spectral regimes^33^. In this work, besides providing results obtained through effective medium theory, we also show the results calculated through the full analytical Mie theory for multilayered isotropic cavities, which agree quite well [see Figs 3 and 4]. It is worth noting that when the loss of silver is considered, as has been done in this work, the anisotropy parameter *η* will be a complicated number. As a result, to obtain the far-field scattering pattern will have to involve the calculation of Bessel and Hankel function of complex orders, where we have implemented in Matlab (The MathWorks, Inc.) through symbolic expression calculation.

To calculate the scattering and absorption spectra, the generalized Mie theory^30^,^37^,^38^,^39^ can be applied for both homogeneous radially anisotropic [Fig. 1(c)] and multilayered isotropic [Fig. 1(b)] spherical cavities [see Eqs (4, 5, 6, 7)]. For the case of multilayered cavities of many layers, we can apply the Cramer’s rule to calculate the scattering coefficients of *a*_*n*_ and *b*_*n*_ based on the coefficients matrix^37^. With *a*_*n*_ and *b*_*n*_ obtained, the far-field scattering intensity Γ is (see refs 4 and 38):


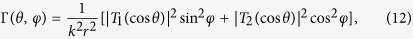


where *r* is the distance between the observation point and the particle center 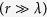, *θ* and *φ* are the polar and azimuthal scattering angles respectively. Here *T*_1,2_ (cos *θ*) can be expressed as:


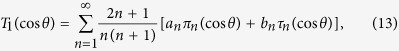



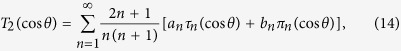


where


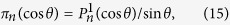



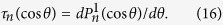


Here 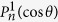 is the associated Legendre function of the first kind of degree *n* and order 1^38^.

## Additional Information

**How to cite this article**: Liu, W. *et al*. Unidirectional superscattering by multilayered cavities of effective radial anisotropy. *Sci. Rep.* **6**, 34775; doi: 10.1038/srep34775 (2016).

## Supplementary Material

Supplementary Information

## References

[b1] Zheludev N. I. & Kivshar Y. S. From metamaterials to metadevices. Nat. Mater. 11, 917–924 (2012). 23089997 10.1038/nmat3431

[b2] Chen H.-T., Taylor A. J. & Yu N. A review of metasurfaces: physics and applications. *arXiv*:*1605*.*07672* (2016). 10.1088/0034-4885/79/7/076401 27308726

[b3] Liu W., Miroshnichenko A. E. & Kivshar Y. S. Control of light scattering by nanoparticles with optically-induced magnetic responses. Chin. Phys. B 23, 047806 (2014).

[b4] Liu W. . Ultra-directional forward scattering by individual core-shell nanoparticles. Opt. Express 22, 16178 (2014). 24977869 10.1364/OE.22.016178

[b5] Jahani S. & Jacob Z. All-dielectric metamaterials. Nat. Nanotech. 11, 23–26 (2016). 10.1038/nnano.2015.304 26740041

[b6] Love A. W. Some highlights in reflector antenna development. Radio. Sci. 11, 671 (1976).

[b7] Jin P. & Ziolkowski R. W. Metamaterial-inspired, electrically small huygens sources. Antennas and Wireless Propagation Letters, IEEE 9, 501 (2010).

[b8] Krasnok A. E., Miroshnichenko A. E., Belov P. A. & Kivshar Y. S. Huygens optical elements and yagi-uda nanoantennas based on dielectric nanoparticles. JETP. Lett. 94, 593 (2011).

[b9] Kerker M., Wang D. S. & Giles C. L. Electromagnetic scattering by magnetic spheres. J. Opt. Soc. Am. 73, 765 (1983).

[b10] Nieto-Vesperinas M., Saenz J. J., Gomez-Medina R. & Chantada L. Optical forces on small magnetodielectric particles. Opt. Express 18, 11428 (2010). 20589003 10.1364/OE.18.011428

[b11] Gomez-Medina R. . Electric and magnetic dipolar response of germanium nanospheres: interference effects, scattering anisotropy, and optical forces. J. Nanophotonics 5, 053512 (2011).

[b12] Liu W., Miroshnichenko A. E., Neshev D. N. & Kivshar Y. S. Broadband unidirectional scattering by magneto-electric core-shell nanoparticles. ACS Nano 6, 5489–5497 (2012). 22545872 10.1021/nn301398a

[b13] Geffrin J. M. . Magnetic and electric coherence in forward- and back-scattered electromagnetic waves by a single dielectric subwavelength sphere. Nat. Commun. 3, 1171 (2012). 23132021 10.1038/ncomms2167

[b14] Filonov D. S. . Experimental verification of the concept of all-dielectric nanoantennas. Appl. Phys. Lett. 100, 201113 (2012).

[b15] Krasnok A. E., Miroshnichenko A. E., Belov P. A. & Kivshar Y. S. All-dielectric optical nanoantennas. Opt. Express 20, 20599 (2012). 23037107 10.1364/OE.20.020599

[b16] Rolly B., Stout B. & Bonod N. Boosting the directivity of optical antennas with magnetic and electric dipolar resonant particles. Opt. Express 20, 20376 (2012). 23037088 10.1364/OE.20.020376

[b17] Fu Y. H., Kuznetsov A. I., Miroshnichenko A. E., Yu Y. F. & Lukyanchuk B. Directional visible light scattering by silicon nanoparticles. Nat. Commun. 4, 1527 (2013). 23443555 10.1038/ncomms2538

[b18] Person S. . Demonstration of zero optical backscattering from single nanoparticles. Nano Lett. 13, 1806 (2013). 23461654 10.1021/nl4005018

[b19] Staude I. . Tailoring directional scattering through magnetic and electric resonances in subwavelength silicon nanodisks. ACS Nano 7, 7824 (2013). 23952969 10.1021/nn402736f

[b20] Hancu I. M., Curto A. G., Castro-Lopez M., Kuttge M. & van Hulst N. F. Multipolar interference for directed light emission. Nano Lett. 14, 166 (2013). 24279805 10.1021/nl403681g

[b21] Vercruysse D. . Unidirectional side scattering of light by a single-element nanoantenna. Nano Lett. 13, 3843–3849 (2013). 23898977 10.1021/nl401877w

[b22] Lukyanchuk B. S., Voshchinnikov N. V., Paniagua-Domnguez R. & Kuznetsov A. I. Optimum forward light scattering by spherical and spheroidal dielectric nanoparticles with high refractive index. ACS Photon. 2, 993–999 (2015).

[b23] Luk’yanchuk B. . The fano resonance in plasmonic nanostructures and metamaterials. Nat. Mater. 9, 707 (2010). 20733610 10.1038/nmat2810

[b24] Liu W. . Scattering of core-shell nanowires with the interference of electric and magnetic resonances. Opt. Lett. 38, 2621–2624 (2013). 23939129 10.1364/OL.38.002621

[b25] Paniagua-Dominguez R. . Generalized brewster effect in dielectric metasurfaces. Nat. Commun. 7, 10362 (2016). 26783075 10.1038/ncomms10362 PMC4735648

[b26] Alu A. & Engheta N. How does zero forward-scattering in magnetodielectric nanoparticles comply with the optical theorem? J. Nanophotonics 4, 041590 (2010).

[b27] Ruan Z. C. & Fan S. H. Design of subwavelength superscattering nanospheres. Appl. Phys. Lett. 98, 043101 (2011).

[b28] Paniagua-Dominguez R., Lopez-Tejeira F., Marques R. & Sanchez-Gil J. A. Metallo-dielectric core-shell nanospheres as building blocks for optical three-dimensional isotropic negative-index metamaterials. New. J. Phys. 13, 123017 (2011).

[b29] Liberal I., Ederra I., Gonzalo R. & Ziolkowski R. W. Induction theorem analysis of resonant nanoparticles: Design of a Huygens source nanoparticle laser. Phys. Rev. Appl. 1, 044002 (2014).

[b30] Liu W. Ultra-directional super-scattering of homogenous spherical particles with radial anisotropy. Opt. Express 23, 14734–14743 (2015). 26072832 10.1364/OE.23.014734

[b31] Stout B., Neviere M. & Popov E. Mie scattering by an anisotropic object. Part 1. homogeneous sphere. J. Opt. Soc. Am. A 23, 1111 (2006). 10.1364/josaa.23.001111 16642189

[b32] Qiu C., Gao L., Joannopoulos J. D. & Soljačić M. Light scattering from anisotropic particles: propagation, localization, and nonlinearity. Laser Photonics Rev. 4, 268 (2010).

[b33] Poddubny A., Iorsh I., Belov P. & Kivshar Y. Hyperbolic metamaterials. Nat. Photon. 7, 948–957 (2013).

[b34] Wu C., Salandrino A., Ni X. & Zhang X. Electrodynamical light trapping using whispering-gallery resonances in hyperbolic cavities. Phys. Rev. X 4, 021015 (2014).

[b35] Liu W., Miroshnichenko A. E. & Kivshar Y. S. Q-factor enhancement in all-dielectric anisotropic nanoresonators. *arXiv*:*1603*.*02111* (2016).

[b36] Liu W., Lei B. & Miroshnichenko A. E. Q-factor and absorption enhancement for plasmonic anisotropic nanoparticles. Opt. Lett. 41, 3563 (2016). 27472619 10.1364/OL.41.003563

[b37] Kerker M. The scattering of light, and other electromagnetic radiation (Academic Press, New York, 1969).

[b38] Bohren C. F. & Huffman D. R. Absorption and Scattering of Light by Small Particles (Wiley, 1983).

[b39] Qiu C. W. & Luk’yanchuk B. Peculiarities in light scattering by spherical particles with radial anisotropy. J. Opt. Soc. Am. A 25, 1623 (2008). 10.1364/josaa.25.001623 18594617

[b40] Johnson P. B. & Christy R. W. Optical constants of the noble metals. Phys. Rev. B 6, 4370 (1972).

[b41] Boardman A. D. Electromagnetic surface modes (Wiley, Chichester; New York, 1982).

[b42] Luo Y., Zhao R. & Pendry J. B. van der Waals interactions at the nanoscale: The effects of nonlocality. PNAS 111, 18422–18427 (2014). 25468982 10.1073/pnas.1420551111 PMC4284608

[b43] Scholl J. A., Koh A. L. & Dionne J. A. Quantum plasmon resonances of individual metallic nanoparticles. Nature 483, 421 (2012). 22437611 10.1038/nature10904

[b44] Savage K. J. . Revealing the quantum regime in tunnelling plasmonics. Nature 491, 574 (2012). 23135399 10.1038/nature11653

[b45] Ruan Z. C. & Fan S. H. Superscattering of light from subwavelength nanostructures. Phys. Rev. Lett. 105, 013901 (2010). 20867445 10.1103/PhysRevLett.105.013901

[b46] Wu D., Jiang S., Cheng Y. & Liu X. Three-layered metallodielectric nanoshells: plausible meta-atoms for metamaterials with isotropic negative refractive index at visible wavelengths. Opt. Express 21, 1076 (2013). 23389001 10.1364/OE.21.001076

[b47] Kuznetsov A. I., Miroshnichenko A. E., Fu Y. H., Zhang J. B. & Lukyanchuk B. S. Magnetic light. Sci. Rep. 2, 492 (2012). 22768382 10.1038/srep00492 PMC3389365

[b48] Evlyukhin A. B. . Demonstration of magnetic dipole resonances of dielectric nanospheres in the visible region. Nano Lett. 12, 3749 (2012). 22703443 10.1021/nl301594s

