# Supplementary material for: Unidirectional superscattering by multilayered cavities of effective radial anisotropy
Source: Sci Rep. 2016 Oct 6;6:34775. doi: 10.1038/srep34775 (PMC5052521; doi:10.1038/srep34775)
Supplement: Supplementary Information [file srep34775-s1.pdf]

Supplementary Information: Unidirectional superscattering by multilayered cavities of effective radial anisotropy

Wei Liu,*, Bing Lei, Jianhua Shi, and Haojun Hu
College of Optoelectronic Science and Engineering, National University of Defense Technology, Changsha, Hunan 410073, China
*Corresponding author: wei.liu.pku@gmail.com

Experimental data for the permittivity of Ag

In our study , we adopt the experimental data from Ref. 1 for the permittivity of silver: $\varepsilon_{Ag}=\varepsilon_{Ag}'+i\varepsilon_{Ag}''$, where ε′ and ε″ corresponds to the real and imaginary part of the permittivity respectively. In Fig. S1, we show the specific data of ε′ and ε″ at the spectral regime of 350-1800 nm.

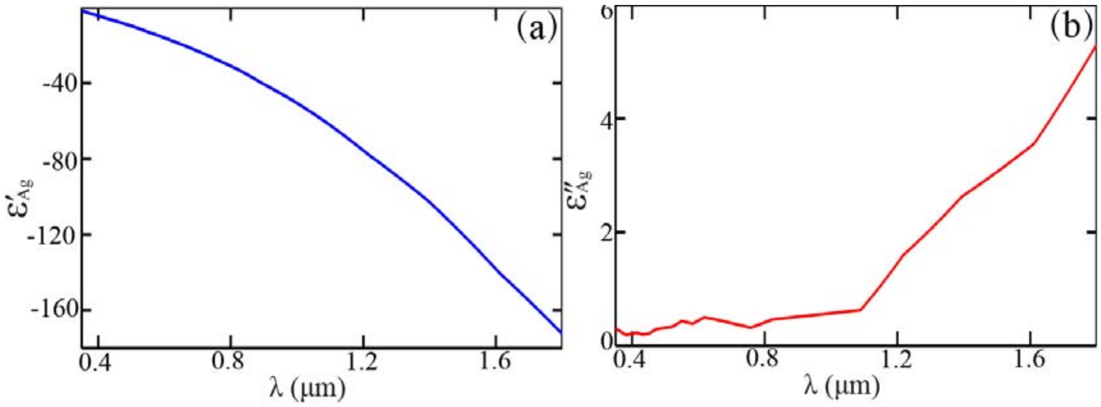

Fig. S1: Experimental data of the real (a) and imaginary (b) part for the permittivity of silver. The data is taken from Ref. 1.

Unidirectional scattering of plasmonic multilayered cavity with the surface scattering effect of thin metal layer considered.

For the permittivity of silver, we can alternatively adopt the Drude model [2,3]:

$$\varepsilon_{\text{D-Ag}}=1-\frac{\omega_{\text{p}}^2}{\omega^2+\text{i}\omega\gamma_d} \quad (1)$$

where ω is the angular frequency of light; ω_p is the plasma frequency and for silver $\omega_p \approx 1.37\times10^{16}$ rad/s; γ_d is the damping rate and can be expressed as[3]:

$$\gamma_d=\gamma_{bulk}+\frac{AV_F}{l_r} \quad (2)$$

where γ_{bulk} is the damping rate for bulk silver and at room temperature we adopt

$\gamma_{bulk}=0.002\omega_p$. The second term on the right hand side of Eq. (2) comes from the surface scattering effect of thin silver layer, where at room temperature A=1, V_F is the Fermi velocity $V_F= 7.37\times10^{-4}\omega_p\lambda_p$. Here λ_p is the plasma wavelength λ_p=137 nm. l_r is the mean free path for electron. For the 14-layered resonator studied in Fig. 3(j), there we have not considered the surface scattering effect of silver. To incorporate such effect, we use the Drude model shown in Eq. (1)-(2) to characterize the permittivity of the thin silver layer, and set l_r =4 nm which is the width of each silver layer. The results are summarized in Fig. S2. Figure S2 (a) shows the scattering and absorption spectra where the resonant position is indicated by point E′ ($\lambda_{E'}$=1545 nm). At this resonant position, the 2D and 3D scattering patterns are shown in Fig. S2 (b) and (c) respectively. It is clear that at this resonant position both ED and MD are efficiently excited. The difference is that the surface scattering effect makes the silver layers more lossy, thus simultaneously enhancing the absorption efficiency and reducing the scattering efficiency. It is worth mentioning that though the loss would reduce the scattering and moreover make the magnitudes of ED and MD different, the coexistence of ED and MD can still guarantee a good directionality of the forward scattering, as is shown in Fig. S2 (b) and (c). In other words, with even the surface scattering effect of thin metal layer considered, the feature of unidirectional scattering has been still preserved.

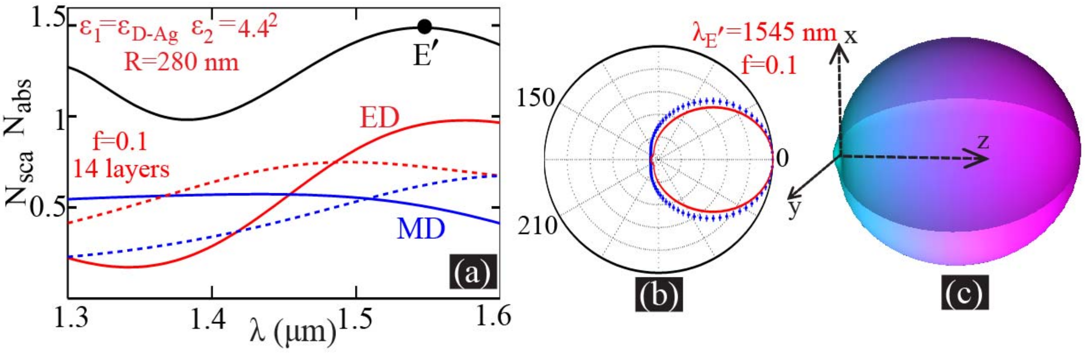

Fig. S2: (a) The scattering and absorption spectra for the multilayered cavity made up of 14 layers with alternating silver [the permittivity is characterized by Eq. (1)-(2), where the surface scattering effect has also been considered] and dielectric ($\varepsilon_2=4.4^2$) layers of width 4 nm and 36 nm respectively. The resonant point is E′ ($\lambda_{E'}$=1545 nm) and at this position the 2D and 3D scattering patterns are shown in (b) and (c) respectively.

References:

1. Johnson, P. B. & Christy, R. W. Optical Constants of the Noble Metals. Phys. Rev. B 6, 4370 (1972).
2. Maier, S. A. Plasmonics: Fundamentals and Applications. (Springer Science & Business Media, 2007).
3. Ruan, Z. & Fan, S. Superscattering of light from subwavelength nanostructures. Physical review letters 105, 13901 (2010).
